# Supplementary figures and images for: Local domestication of lactic acid bacteria via cassava beer fermentation
Source: PeerJ. 2014 Jul 8;2:e479. doi: 10.7717/peerj.479 (PMC4103073; doi:10.7717/peerj.479)

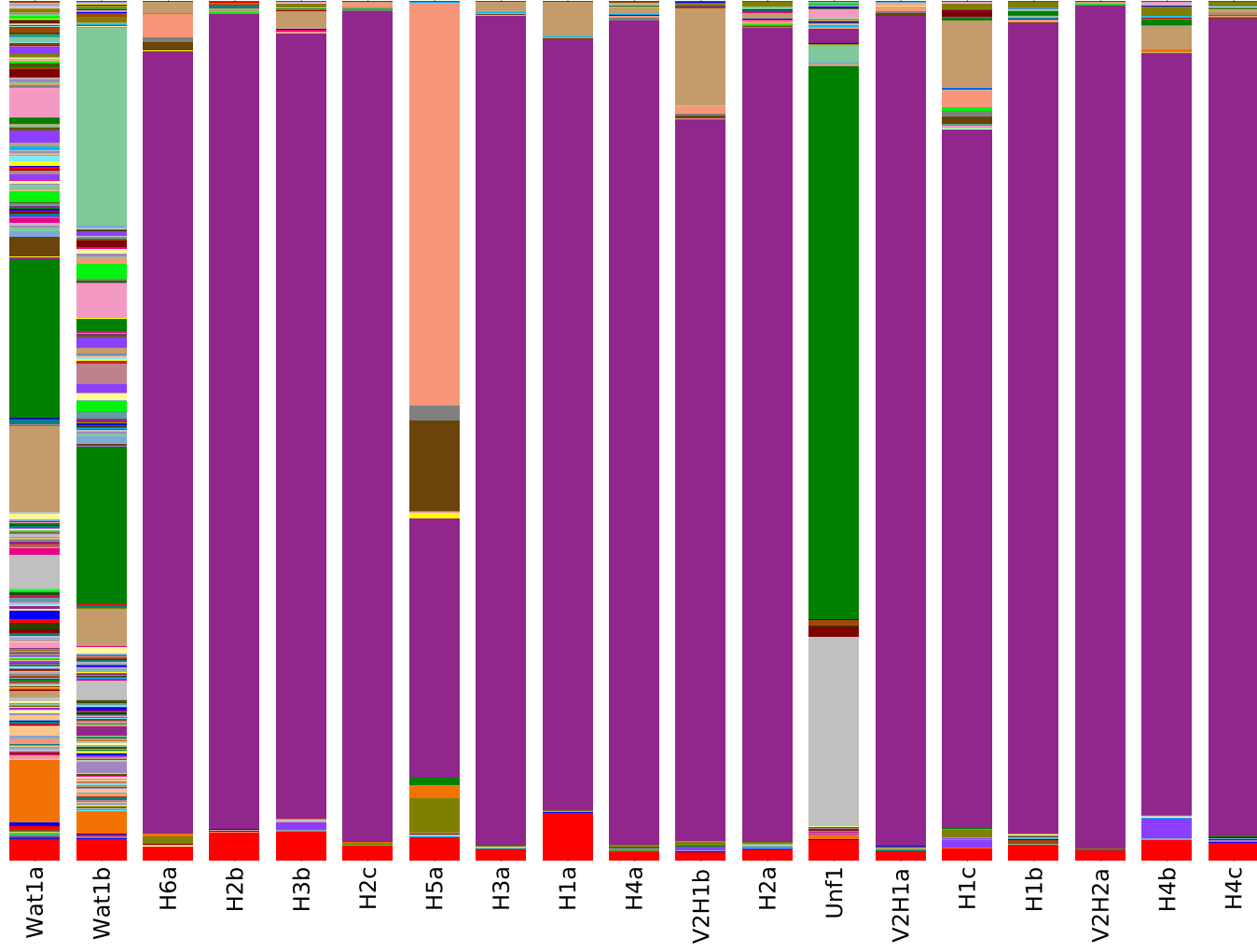

Supplement: Figure S1 — Wat1 – water sampled from village 1 H1 – houses 1–6, time points a-cV2H1a – Village 2; houses 1–2, time points a-b Unf1 – Unfermented sample from village 2 Note: Samples H6a and H5a were excluded during analysis due to insufficient OTU count. See “Supplemental Legend” file for information on OTU taxonomic classification. [file peerj-02-479-s001.pdf]
